# MedicalPatchNet: a patch-based self-explainable AI architecture for chest X-ray classification

**DOI:** 10.1038/s41598-026-40358-0

**Published:** 2026-02-20

**Authors:** Patrick Wienholt, Christiane Kuhl, Jakob Nikolas Kather, Sven Nebelung, Daniel Truhn

**Affiliations:** 1 Department of Diagnostic and Interventional Radiology, University Hospital RWTH Aachen, Aachen, Germany; 2 Technical University Dresden, Else Kroener Fresenius Center for Digital Health, Dresden, Germany; 3 Department of Medicine III, University Hospital RWTH Aachen, Aachen, Germany; 4 Pathology & Data Analytics, Leeds Institute of Medical Research at St James’s,University of Leeds, Leeds, United Kingdom; 5 Department of Medicine I, University Hospital Dresden, Dresden, Germany; 6 Medical Oncology, National Center for Tumor Diseases (NCT), University Hospital Heidelberg, Heidelberg, Germany

**Keywords:** Computational biology and bioinformatics, Health care, Mathematics and computing, Medical research

## Abstract

Deep neural networks excel in radiological image classification but frequently suffer from poor interpretability, limiting clinical acceptance. We present MedicalPatchNet, an inherently self-explainable architecture for chest X-ray classification that transparently attributes decisions to distinct image regions. MedicalPatchNet splits images into non-overlapping patches, independently classifies each patch, and aggregates predictions, enabling intuitive visualization of each patch’s diagnostic contribution without post-hoc techniques. Trained on the CheXpert dataset (223,414 images), MedicalPatchNet matches the classification performance (AUROC 0.907 vs. 0.908) of EfficientNetV2-S, while improving interpretability: MedicalPatchNet demonstrates improved interpretability with higher pathology localization accuracy (mean hit-rate 0.485 vs. 0.376 with Grad-CAM) on the CheXlocalize dataset. By providing explicit, reliable explanations accessible even to non-AI experts, MedicalPatchNet mitigates risks associated with shortcut learning, thus improving clinical trust. Our model is publicly available with reproducible training and inference scripts and contributes to safer, explainable AI-assisted diagnostics across medical imaging domains. We make the code publicly available: https://github.com/TruhnLab/MedicalPatchNet

## Introduction

Neural networks are increasingly integrated into clinical workflows^1^. In particular for radiological tasks they have shown remarkable results. Applications range from breast cancer screening^2^, where neural networks can identify suspicious cases earlier than human experts, over mortality prediction in intensive care units^3^, and diagnosing common diseases from chest X-rays, where deep learning models have consistently proven beneficial. Similar methodological trends can also be observed in a range of recent studies on machine learning and medical data analysis^4–12^.

Despite their strong and sometimes superior performance compared to expert radiologists^13^, these models often lack explainability regarding their decision-making process^14^. Providing explanations is crucial for correctly interpreting the results, especially in cases where the network’s behavior differs from the radiologist’s expectations. Because neural networks often rely on different image features than humans^15^, knowing the underlying reasons for a decision is particularly important. Without explainability, a neural network with superior performance is of reduced value, as a medical professional may be unable to determine the reasoning behind the decision, making it difficult to assess its trustworthiness. Furthermore, explanations provided by neural networks should be easily comprehensible, especially for the people using them, not just for the people designing them. To be practically useful in clinical settings, individuals without extensive deep learning knowledge should be able to easily and intuitively interpret the decisions.

However, most modern deep learning architectures have been designed primarily to maximize performance rather than explainability. This results in architectures that operate as so-called black boxes, making it difficult to gain insight into them^14^. Nevertheless, since explainability is essential, methods have been developed to render these black box approaches post-hoc explainable. Examples include Grad-CAM^16^, GradCam++^17^, Eigen-CAM^18^, and other methods^19–22^. These techniques are widely utilized in medical contexts^23^, as they generate saliency maps that attempt to shed light on the underlying decision process. However, post-hoc explainability methods have inherent limitations^24^. Previous research has shown that common saliency methods like Grad-CAM do not necessarily highlight the most critical regions influencing the decision^25,26^.

Correctly interpreting results of methods like Grad-CAM requires deep understanding of gradient-based mechanisms in deep learning, a level of knowledge beyond what is typically expected from clinical personnel who should routinely use these networks. Depending on these methods, which produce visually appealing but potentially misinterpreted saliency maps, may create a false sense of comprehension regarding the network’s decision-making process. Consequently, this misplaced trust could lead to relying on predictions that are not truly trustworthy. Rudin et al.^24^ therefore argues against using these post-hoc methods, as they produce misleading and unreliable explanations, and instead advocate for inherently explainable approaches. Self-explainable AI refers to designing AI that is explainable by design^24,27^. One approach is to learn, for each class, a set of representative image patches that capture characteristic visual patterns, and then classify new images by comparing their local regions to these class-specific prototypes and aggregating the resulting evidence^28^.

A non-gradient-based approach is to occlude parts of the image and then evaluate the change in the output compared to the non-occluded image^22^. To make brain MRI scan decisions explainable, Zhang et al.^29^ developed a method that identifies and extracts the most discriminative 3D patches. Their network then utilizes only these patches, allowing the decision to be known to rely exclusively on them, rather than on other patches. Additionally, to interpret model predictions, patches are perturbed by zeroing them out, and the network’s behavior is observed. This allows for quantifying the respective contributions. Previous studies have used different patch-based approaches to address classification tasks. For instance, Oh et al.^30^ and Szczepanski et al.^31^ classified COVID-19 by extracting randomly overlapping image patches, training a model on these patches individually rather than on the entire image. This patch-wise approach was shown to effectively reduce overfitting, allowing more efficient training on limited datasets. However, their interpretability depended on applying Grad-CAM^16^ to each patch individually and aggregating the resulting saliency maps for visualization, thereby inheriting the limitations of post-hoc methods.

In recent work, several Multiple Instance Learning (MIL) frameworks^32–34^ similarly divide images into patches and aggregate patch-level evidence. Ilse et al.^32^ propose attention-based MIL, learning permutation-invariant attention weights to identify diagnostically relevant instances while preserving interpretability. Lu et al.^33^ introduce CLAM, which combines attention pooling with instance-level clustering to refine feature separation and generate high-contrast heatmaps under weak supervision. Shao et al.^34^ present TransMIL, a transformer-based correlated MIL approach that models spatial and morphological relationships between patches to improve performance and visual interpretability.

A related field that partially overlaps with explainability is weakly supervised segmentation^35,36^. In this approach, segmentation masks are derived solely from classification labels. Techniques such as Grad-CAM^16^ and attention maps^37^ are frequently employed in this context. Unlike explainability techniques, the primary goal of this field is to produce accurate segmentation masks, without necessarily focusing on transparency regarding biases or underlying decision mechanisms. This stands in contrast to explainability methods, whose focus is to reflect the true underlying decision process of the neural network. An explainability method that correctly segments a pleural effusion, for example, but does so despite the true underlying reason for classification being elsewhere, is a poor explainability method–even if the segmentation of the pathology is correct. It has been shown that for chest X-rays, networks tend to rely on dataset biases when classifying pathologies^38,39^. Zech et al., for example, show that their network sometimes relied on the presence of specific laterality markers—small radiopaque “L” or “R” labels that indicate the patient’s left or right side on the radiograph—to detect pneumonia^38^. Similarly, DeGrave et al.^39^ demonstrated that some networks trained for COVID-19 classification relied on shortcuts–such as laterality markers or radiopacity at image borders–rather than on genuine pathological features.

All these factors highlight the need for a neural network for clinical applications that combines high performance, self-explainability, and ease of interpretation, even for users without extensive deep learning expertise. This is precisely where our method, MedicalPatchNet, stands out. We introduce MedicalPatchNet, which demonstrates strong performance in chest X-ray classification. Instead of complex interpretation techniques that require advanced insight into a model’s inner workings, our approach is straightforward yet effective: MedicalPatchNet splits the input image into patches, classifies each patch independently, and determines the final diagnosis by averaging these patch-level predictions. Visualizing the classification results at the patch level clearly illustrates each patch’s contribution to the overall decision. This explicit visualization ensures that every influential visual feature is transparently represented. Our architecture can be viewed as a special case of the Multiple Instance Learning frameworks prevalent in computational pathology. In contrast to such approaches, which often rely on complex, learnable aggregation functions like attention mechanisms, MedicalPatchNet employs a fixed, non-learnable arithmetic mean of the patch logits. This simplification makes our model a more constrained but inherently transparent variant, where each patch’s contribution to the final decision is explicitly defined and not learned.

Our proposed architecture can accommodate various backbone architectures. For our study, we chose EfficientNetV2-S^40^, as it provides good performance with relatively low computational requirements. First, we demonstrate that MedicalPatchNet with an EfficientNetV2-S backbone achieves performance comparable to the standard EfficientNetV2-S architecture for classification of the 14 classes on the CheXpert^41^ dataset. Maintaining competitive performance is crucial, as explainability does not necessarily correlate with diagnostic accuracy^24^. Second, we evaluate the saliency maps generated by MedicalPatchNet on the CheXlocalize dataset^25^ (see Fig. 1), comparing its localization capabilities against Grad-CAM, Grad-CAM++, and Eigen-CAM using the EfficientNetV2-S. Here, MedicalPatchNet outperforms these three commonly used explainability methods.

## Material and methods

**Fig. 1:**
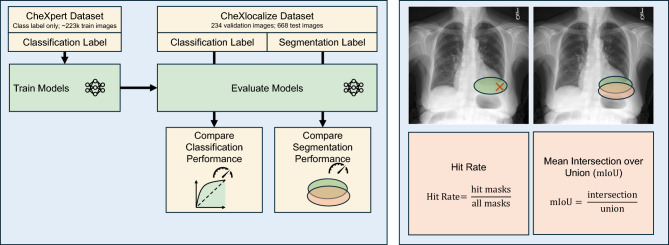
Evaluation framework for classification and localization. Models are trained on the CheXpert dataset using only classification labels. For evaluation, the CheXlocalize dataset is used, which provides ground-truth segmentation masks. We compare both classification performance (e.g., using AUROC) and localization performance. For localization, saliency maps from our proposed MedicalPatchNet are compared against a standard EfficientNet-B0 explained by post-hoc methods (Grad-CAM, Grad-CAM++, and Eigen-CAM). The primary localization metrics, illustrated on the right, are the Hit Rate and the Mean Intersection over Union (mIoU).

### Material: datasets and annotations

Our study is based on the CheXpert dataset^41^, labeled by the VisualCheXpert tool^42^, which provides the large-scale chest X-ray material and image-level annotations used throughout our experiments. The training dataset comprised 223,414 chest X-rays from 64,540 patients, each labeled with binary classifications across $C = 14$ distinct classes. The CheXpert label set includes No Finding, Lung Opacity, Lung Lesion, Edema, Consolidation, Pneumonia, Atelectasis, Pneumothorax, Pleural Effusion, Pleural Other, Cardiomegaly, Enlarged Cardiomediastinum, Fracture, and Support Devices. All radiographs were preprocessed once before training by cropping them to a square field of view and isotropically resizing them to $1024 \times 1024$ pixels. For all experiments, we used the image-level labels provided by the VisualCheXpert^42^ tool. Of these training images, 191,027 have a frontal projection, while 32,387 are lateral views. The frontal and lateral views are not combined or used together but are treated as two separate images for both training and evaluation. To evaluate how effectively the methods can localize pathologies, we use the CheXlocalize dataset^25^, which consists of a validation set comprising 234 chest X-rays from 200 patients and a test set containing 668 chest X-rays from 500 patients. These images are distinct from the CheXpert training data; they correspond to CheXpert validation and test sets enhanced with segmentation masks annotated by radiologists. For 10 of the 14 CheXpert labels (Lung Opacity, Atelectasis, Cardiomegaly, Consolidation, Edema, Enlarged Cardiomediastinum, Lung Lesion, Pleural Effusion, Pneumothorax, and Support Devices), CheXlocalize provides pixel-wise segmentation masks, which we later use to quantify localization performance.

### MedicalPatchNet

**Fig. 2:**
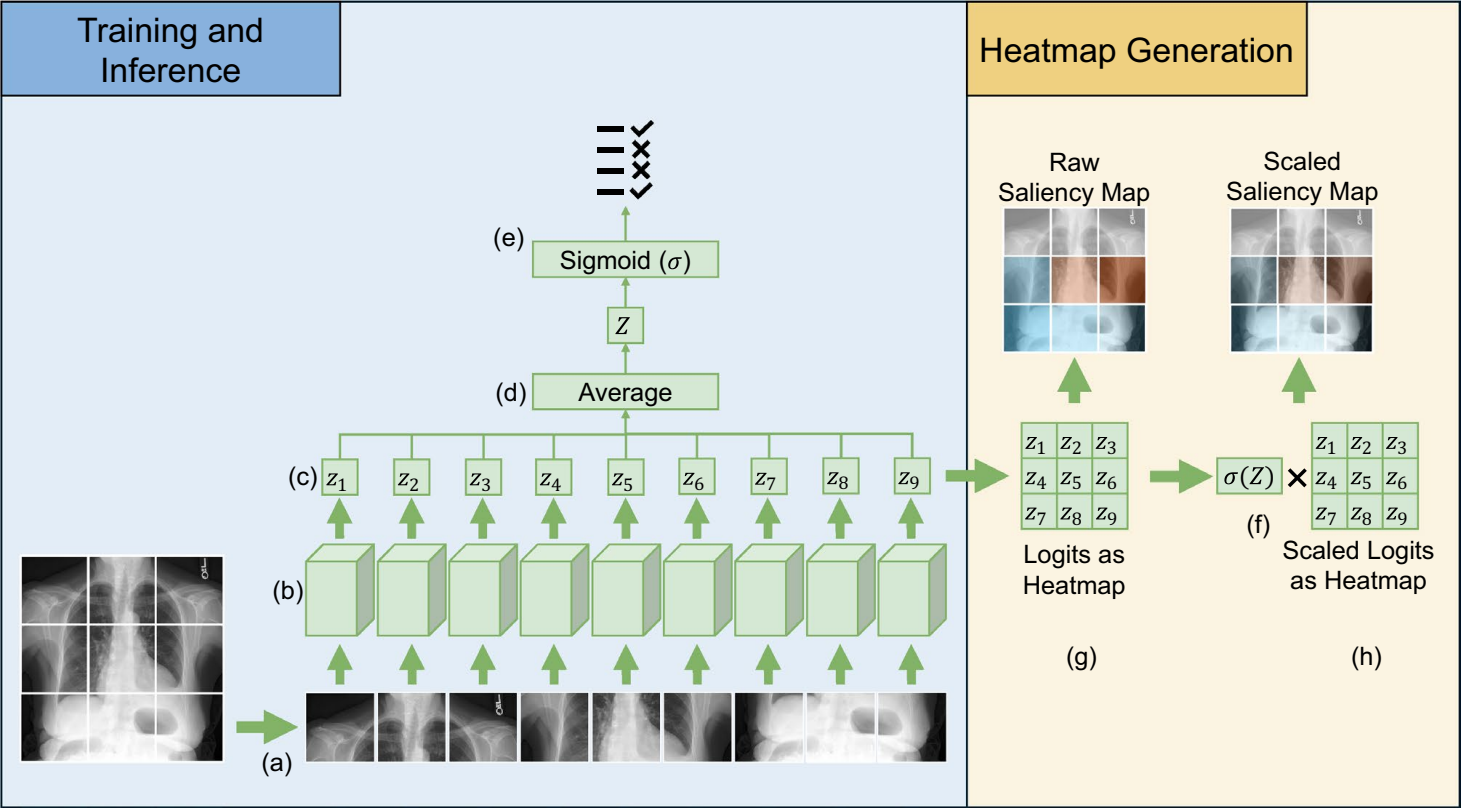
Initially, the image is divided into patches (**a**), each patch is independently processed by the same EfficientNetV2-S (**b**). The resulting patch logits, i.e., raw pre-sigmoid class scores (**c**), are averaged (**d**). After applying the sigmoid activation function, the output (**e**) provides the final classification results of MedicalPatchNet. Multiplying the raw patch logits by the classification probabilities generates scaled patch logits (**f**). A saliency map can be derived either from raw patch logits (**g**) or scaled patch logits (**h**), illustrating each patch’s contribution to the final decision.

The core idea of MedicalPatchNet, as shown in Fig. 2, is to divide the image into patches, predict each patch independently of the others, and then combine the predictions into a global prediction. Since the global prediction is just a scaled sum of the local predictions, visualizing these local predictions allows direct quantification of each patch’s contribution to the final classification. By construction, all patches are processed independently with a shared backbone and their logits are combined by a fixed arithmetic mean, without any learnable interaction between patches. This simple aggregation strategy allows MedicalPatchNet to act as a drop-in replacement for a conventional image-level classifier that uses the same backbone and training labels, while additionally providing an explicit patch-wise decomposition of the decision.

As a backbone, we chose EfficientNetV2-S^40^; however, other architectures are also compatible. Since the chest X-rays are grayscale, we adapt the backbone network to accept a single input channel instead of three by modifying the first convolution layer. As a first step, MedicalPatchNet partitions an input image of size $S \times S$ pixels into $P \times P$ non-overlapping patches, each sized $p \times p$ pixels, where $p = \frac{S}{P}$. These patches $x_i$, for $i \in [1,P^2]$, are stacked along the batch dimension for processing. Thus, a batch containing *B* images results in a batch size of $BP^2$ patches, all processed simultaneously by the backbone neural network. We modify the final layer to output *C* logits, i.e. raw, pre-sigmoid scores for each class, producing an output vector $z_i \in \mathbb {R}^{C}$ for each patch. It contains one logit for each class. The global logits $Z \in \mathbb {R}^{C}$ are computed by averaging these local logits: $Z = \frac{1}{P^2}\sum _{i}^{P^2}z_i$. The final classification output is obtained by applying the sigmoid function: $\hat{y} = \sigma (Z)$. The idea of applying the sigmoid after averaging the logits is to avoid constraining the patch logits’ influence on the global logits.

Using the raw logits vector $z_i$ and the classification vector $\hat{y}$, we calculate scaled patch logits as $\hat{z}_i = \hat{y} \odot z_i$ through element-wise multiplication. By scaling the raw logits with classification probabilities, we incorporate a limited degree of global context into individual patches in an interpretable manner.

**Fig. 3:**
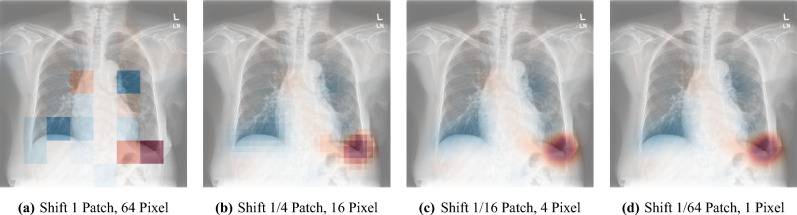
The saliency map shown in (**a**) illustrates the influence of each patch on the final classification of pleural effusion. Patches with large positive logits are shown in red and represent strong evidence supporting the class, patches with large negative logits are shown in blue and represent evidence against the class, and patches visualized in light grey or white have logits close to zero and therefore contribute only minimally to the final decision (effectively “abstaining” from the vote). Image (**a**) shows a direct visualization of the patch logits from one forward pass. When shifting the image and averaging the generated saliency maps, smoother maps can be produced, as seen in (**b**), (**c**), and (**d**), although this requires more forward passes.

Saliency maps derived from raw or scaled logits are generated in the same manner. To create a coarse saliency map as illustrated in Fig. 3a, we visualize the patch logits as an overlay saliency map. The saliency map values can be interpreted as follows: a high positive value indicates that the network strongly “decides” the entire image belongs to the specific class based exclusively on the content of that patch. Conversely, a high negative value signifies that, based solely on the patch, the network strongly “decides” against classification into that class. Patches that are irrelevant to the classification decision have values close to zero. Computing global logits by averaging local patch logits can thus be viewed as patches “voting” for or against the presence of a class, with different strength.

To improve the coarse appearance of patch-based saliency maps, we generate multiple spatially shifted saliency maps and overlay them. First, we define a shift offset *o*, which determines the stride of the image shifts used for additional saliency map generation. Given a patch size *p*, an offset *o* results in $\left( \frac{p}{o}\right) ^2$ total shifts, since shifts of size *o* are performed along both spatial dimensions. Image borders are padded with zero values. The smoothed saliency map is obtained by averaging all shifted patch saliency maps, which reduces block artifacts. The shift offset *o* is adjustable as a hyperparameter and directly trades off explanation resolution and computational cost. An offset of $o = p$ generates only one saliency map, equivalent to direct patch prediction, so that each of the original non-overlapping image patches is evaluated exactly once. Conversely, setting the offset to $o = 1$ results in pixel-wise shifts, necessitating $p^2$ forward passes to produce the saliency map shown in 3d. More generally, for a square image of size $S \times S$, the number of evaluated patches, and thus the explanation runtime, scales proportionally to $\left( \frac{S}{o}\right) ^2$. It should be noted that MedicalPatchNet with a patch size equal to the image size generates only one output vector, representing the entire image. Thus, using this configuration is equivalent to a standard EfficientNetV2-S image-level classifier; only the first convolutional and final linear layers are adapted to our data (single-channel input and the chosen number of output classes).

### Training

We trained both MedicalPatchNet and EfficientNetV2-S on chest X-rays rescaled to $512 \times 512$ pixels. Concretely, all images were first preprocessed to a square format of $1024 \times 1024$ pixels as described above and, at train and test time, were then resized to $512 \times 512$ pixels. MedicalPatchNet divides each image into $8 \times 8$ patches, each measuring $64 \times 64$ pixels. We initialized EfficientNetV2-S, both standalone and as the backbone for MedicalPatchNet, with pretrained weights from ImageNet-1k^43^. All models were trained exclusively on classification labels from the CheXpert training dataset^41^, generated using the VisualCheXpert labeling tool^42^. During training, no segmentation masks or explicit localization information were provided. The loss was computed only for the global classification output and backpropagated through the entire network.

We trained the models using PyTorch version 2.5.1 on Nvidia H100 GPUs. We set the batch size to 16 images. For MedicalPatchNet, this corresponded to an effective batch size of $16 \times 8 \times 8 = 1024$ patches, as all patches from one image were processed simultaneously through the backbone network. As an optimizer, we used AdamW^44^ and trained for 20 epochs with a OneCycle learning rate schedule^45^, peaking at a learning rate of $1 \cdot 10^{-4}$ after the first 5% of training. The complete training of a single model required approximately four hours. Image augmentation involved randomly cropping a square area covering between 50% and 100% of the original image, followed by random rotations within $\pm 5^{\circ }$ and brightness adjustments with a factor between 0.7 and 1.3. All images were loaded as single-channel grayscale, converted to tensors, and intensity values were scaled to the range [0, 1]; no additional per-image standardization or histogram equalization was applied.

### Evaluation

To confirm that dividing images into patches did not negatively impact classification performance, we compared the performance of MedicalPatchNet against EfficientNetV2-S. Because both models share the same backbone, training data, and optimization pipeline, this comparison effectively isolates the impact of enforcing patch-wise independence with fixed averaging aggregation versus standard image-level classification on overall diagnostic performance. As performance metrics, we used sensitivity, specificity, accuracy, and the Area Under the Receiver Operating Characteristic curve (AUROC). Sensitivity (true positive rate) measures the proportion of correctly identified positives, while specificity (true negative rate) measures the proportion of correctly identified negative cases. Accuracy is the overall proportion of correctly classified instances. AUROC quantifies the model’s ability to distinguish between classes by summarizing its performance across all possible classification thresholds, with a higher AUROC indicating better class separability. An AUROC of 0.5 represents a random classifier. For sensitivity, specificity, and accuracy, optimal classification thresholds were selected for each class by maximizing the sum of sensitivity and specificity on the validation dataset. These thresholds were then used during evaluation on the held-out test dataset. We estimated 95% confidence intervals for performance metrics using bootstrapping, resampling the test dataset with replacement 100,000 times.

We evaluated the localization performance of the saliency maps produced by MedicalPatchNet and those generated by Grad-CAM, Grad-CAM++, and Eigen-CAM applied to EfficientNetV2-S, using the CheXlocalize dataset^25^.

We used CheXlocalize purely as an external evaluation set; all model parameters were learned exclusively from the CheXpert training subset.

In addition to these post-hoc baselines, we also trained two prototype-based, inherently interpretable methods (ProtoPNet^28^ and PIPNet^46^) using the authors’ public reference implementations and their default training hyperparameters on CheXpert. In our setting, however, both approaches failed to yield a reliable 14-label multi-label chest X-ray classifier (ProtoPNet remained close to chance level, and PIPNet collapsed to meaningful predictions for only a small subset of labels). Because meaningful explanations require a non-trivial classifier, we do not treat these models as competitive localization baselines in the main text; instead, we provide the complete quantitative results and a detailed discussion of possible reasons for this failure in the Supplementary Material.

We evaluated the saliency maps based on their hit rate, defined as follows^25^: For each ground-truth segmentation mask, we identify the pixel with the maximum saliency value. If this pixel lies within the corresponding ground-truth segmentation mask, we count a hit; otherwise, we count a miss. Let $h_i \in \{0,1\}$ denote the hit indicator for case *i* and let *N* be the number of evaluated cases. The hit rate is then defined as

$$\begin{aligned} \text {hit rate} = \frac{1}{N} \sum _{i=1}^{N} h_i. \end{aligned}$$

Intuitively, the hit rate measures how often the single most highlighted point of a saliency map falls inside the expert-annotated pathology region, independent of the exact size or shape of that region.

Additionally, we computed the mean Intersection over Union (mIoU), defined as the average of the intersection areas between predicted saliency maps and ground truth segmentations, divided by their union, calculated across all true positive, false positive, and false negative cases. In this context, a true positive (TP) case denotes an image–pathology pair with a positive ground-truth label and a non-empty predicted segmentation mask after thresholding; a false positive (FP) has a non-empty predicted mask but a negative ground-truth label; a false negative (FN) has a positive ground-truth label but no predicted mask; and a true negative (TN) has a negative ground-truth label and no predicted mask. This metric quantifies the spatial overlap quality of the saliency maps with respect to expert-annotated pathology regions. In contrast to the hit rate, which only checks whether the most salient point lies inside the lesion, mIoU is sensitive to the full extent and shape of the highlighted area and penalizes overly diffuse or misplaced saliency. For the saliency-based localization metrics (hit rate and mIoU), we used the official CheXlocalize evaluation code^25^ with its default bootstrapping configuration to compute 95% confidence intervals.

Unlike Saporta et al.^25^, our mIoU evaluation included not only true positive cases–where both saliency and ground truth segmentation exist–but also evaluated false positive and false negative cases. To convert continuous saliency maps into binary segmentation masks, we tuned a single threshold per pathology on the CheXlocalize validation set by maximizing the mean Intersection over Union with respect to the radiologist-annotated ground-truth pixel-level masks provided as part of the CheXlocalize dataset (Tables 1 and 2).

## Results

**Table 1:** Performance comparison between MedicalPatchNet and EfficientNetV2-S. The metrics represent average values computed across the 10 classes from the CheXlocalize dataset (*) and across all 14 classes from the CheXpert dataset (All).

Model	AUROC		Accuracy		Sensitivity		Specificity	
	*	All	*	All	*	All	*	All
MedicalPatchNet	0.907	0.902	**0.836**	**0.848**	0.825	0.763	**0.841**	**0.851**
EfficientNetV2-S	**0.908**	**0.911**	0.823	0.843	**0.834**	**0.798**	0.826	0.844
Difference	−0.001	−0.009	0.013	0.005	−0.009	−0.035	0.015	0.007

**Fig. 4:**
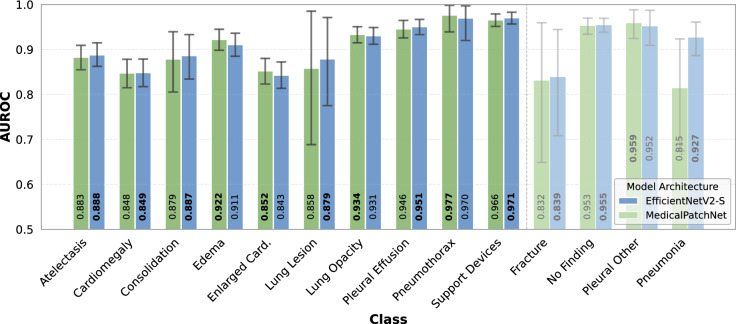
Comparison of the Area Under the Receiver Operating Characteristic (AUROC) curves for MedicalPatchNet and EfficientNetV2-S, indicating similar classification performance.On the CheXlocalize dataset^25^, both models yield mean AUROCs of 0.907 and 0.908, respectively; for the full 14-class CheXpert dataset^41^, AUROCs are 0.902 and 0.911, respectively.

Table 1 presents the performance comparison between MedicalPatchNet and EfficientNetV2-S. It provides averaged metrics across all classes, as well as specifically across the 10 classes included in the CheXlocalize dataset. Figure 4 displays the AUROC scores for each individual class. Detailed accuracy, sensitivity, and specificity values for all classes, as well as an additional backbone generalizability experiment comparing MedicalPatchNet–ConvNeXtBase to a standard ConvNeXt^47^ classifier, are provided in the Supplementary Material. To assess how effectively each saliency method identifies pathologies, we evaluated the hit rate, which is summarized in Table 3. For each pathology, this table reports the proportion of cases in which the single most salient point of a method’s map falls inside the corresponding expert-annotated segmentation mask (values between 0 and 1, higher is better). As expected, the radiologist benchmark substantially exceeds all automatic methods (mean hit rate 0.814 versus 0.471 and 0.485 for scaled and raw MedicalPatchNet encodings). MedicalPatchNet demonstrates superior performance in nine out of the ten analyzed classes. Scaled patch saliency maps outperform other methods in six classes, while raw patch saliency maps perform best in three classes. The average mean intersection over union (mIoU), computed across true positives, false positives, and false negatives, is presented in Table 2; MedicalPatchNet shows the highest overall performance. However, when evaluating mIoU exclusively for true positive cases, the best performance is achieved by Grad-CAM++. From a clinical perspective, the aggregate mIoU over true positives, false positives, and false negatives is particularly relevant, because it also penalizes saliency methods that highlight incorrect regions in false positive cases or fail to localize pathology in false negative cases, i.e. precisely in situations where reliable visual feedback is most critical.

**Table 2:** Mean intersection over union (mIoU) averaged across true positives (TP), false positives (FP), and false negatives (FN), and reported separately for true positives (TP only).

Pathology	MedicalPatchNet		EfficientNetV2-S		
	Scaled Patch Saliency	Raw Patch Saliency	Grad-CAM	Grad-CAM++	Eigen-CAM
TP, FP, FN	**0.069**	0.056	0.052	0.054	0.049
TP only	0.168	0.164	0.227	**0.238**	0.215

**Table 3:** Hit rate of different saliency methods evaluated on the CheXlocalize dataset. Bold indicates the highest score, and underlining indicates the second-best score for each pathology. The 95% confidence intervals are presented in brackets. The human benchmark values are obtained from a radiologist and are reported by Saporta et al.^25^.

Pathology	MedicalPatchNet		EfficientNetV2-S			Human
	Scaled Patch Saliency	Raw Patch Saliency	Grad-CAM	Grad-CAM++	Eigen-CAM	
Lung Opacity	**0.493** [0.438–0.548]	0.395 [0.339–0.451]	0.424 [0.369–0.481]	0.414 [0.359–0.470]	0.376 [0.323–0.430]	0.559
Atelectasis	**0.490** [0.413–0.567]	0.389 [0.315–0.463]	0.406 [0.335–0.483]	0.367 [0.296–0.440]	0.373 [0.301–0.451]	0.870
Cardiomegaly	**0.464** [0.390–0.535]	0.269 [0.206–0.339]	0.376 [0.305–0.446]	0.246 [0.184–0.311]	0.217 [0.157–0.278]	0.972
Consolidation	**0.540** [0.381–0.706]	0.482 [0.323–0.643]	0.516 [0.361–0.688]	0.401 [0.250–0.568]	0.433 [0.265–0.600]	0.510
Edema	0.648 [0.543–0.750]	0.601 [0.488–0.707]	**0.650** [0.545–0.747]	0.471 [0.367–0.573]	0.518 [0.407–0.634]	0.769
Enlarged Card.	**0.419** [0.361–0.472]	0.320 [0.266–0.374]	0.392 [0.340–0.448]	0.349 [0.294–0.403]	0.322 [0.267–0.374]	0.957
Lung Lesion	0.281 [0.062–0.545]	**0.564** [0.267–0.824]	0.216 [0.000–0.444]	0.284 [0.071–0.538]	0.354 [0.100–0.615]	0.850
Pleural Effusion	**0.466** [0.378–0.552]	0.372 [0.280–0.458]	0.308 [0.226–0.386]	0.293 [0.213–0.375]	0.207 [0.134–0.283]	0.718
Pneumothorax	0.498 [0.182–0.833]	**0.794** [0.500–1.000]	0.195 [0.000–0.500]	0.195 [0.000–0.500]	0.097 [0.000–0.333]	1.000
Support Devices	0.409 [0.353–0.462]	**0.668** [0.614–0.717]	0.272 [0.223–0.316]	0.225 [0.179–0.268]	0.130 [0.093–0.169]	0.933
Mean	0.471	**0.485**	0.376	0.325	0.303	0.814

**Fig. 5:**
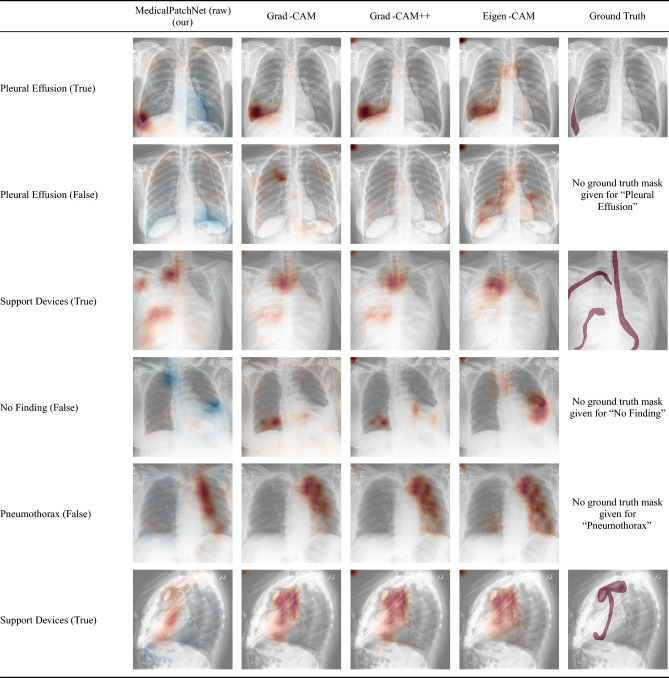
Representative saliency maps produced by MedicalPatchNet and three post-hoc methods. Each row displays the same chest X-ray for a given pathology together with its ground-truth label (“True” or “False”). Columns compare MedicalPatchNet’s raw patch logits with Grad-CAM, Grad-CAM++, and Eigen-CAM applied to an EfficientNetV2-S baseline. In the MedicalPatchNet maps, **red** denotes evidence supporting the class and *blue* denotes evidence against it, whereas Grad-CAM–based maps visualize only positive (red) contributions. Eigen-CAM is class-agnostic and therefore does not generate class-specific saliency maps. Interestingly, for the wrongly diagnosed pneumothorax, all four explainability methods point to the chest tube, revealing that the model used a shortcut, with MedicalPatchnet denoting its course most clearly.

## Discussion

The results presented in Table 1 demonstrate that MedicalPatchNet, using an image size of $512 \times 512$ and a patch size of $64 \times 64$, achieves classification performance comparable to EfficientNetV2-S, which utilizes the full $512 \times 512$ context. The results show that the classification performance of MedicalPatchNet matches that of EfficientNetV2-S, except for pneumonia. This indicates that classification of these chest X-ray pathologies can be achieved with only local information. It is not necessary for the different patches and their processing to communicate until the logits are averaged at the end. Importantly, the aim of MedicalPatchNet is not to surpass the original EfficientNetV2-S in raw classification accuracy, but to retain comparable performance under the additional structural constraint that all evidence is decomposed into local, independently processed patches. In this sense, matching the backbone’s AUROC demonstrates that a substantially more constrained and interpretable aggregation scheme can be imposed without incurring a large loss in diagnostic performance. A complementary experiment on natural images (PASCAL VOC 2012, Supplementary Material, section “Evaluation on natural images”) shows that the AUROC gap between MedicalPatchNet and EfficientNetV2-S is slightly larger on complex multi-object scenes, which is consistent with the intuition that tasks requiring stronger global context benefit more from a fully image-level architecture. However, it should be noted that this observation is limited to the pathologies examined here, as one can imagine cases solvable by EfficientNetV2-S but not by MedicalPatchNet, as discussed in the limitations. We further examined the impact of patch size on classification performance by training MedicalPatchNet with patch sizes of $64\times 64$, $128\times 128$, and $256\times 256$, and comparing it to an image-level EfficientNetV2-S (effectively a single $512\times 512$ patch). On average across all pathologies, the mean AUROC varied only marginally between these configurations (approximately 0.900–0.911), although for some individual findings, such as pneumonia, larger patches yielded noticeably higher AUROC than smaller ones. Detailed per-class results are reported in the Supplementary Material.

Having reliable explainability methods is essential for the clinical application of neural networks, as these provide insights into their decision processes. Especially when they make decisions that contradict human reasoning or appear unintuitive at first glance. For example, if the network learns to classify pathologies based on features that correlate in the dataset but are unrelated in reality, a reliable explainability method allows investigation of such behavior. DeGrave et al.^39^ demonstrated that some networks trained for COVID-19 classification rely on shortcuts–such as laterality markers or radiopacity at image borders–rather than on genuine pathological features. A similar observation was made by Zech et al.^38^ who discovered that pneumonia classification sometimes relied on laterality markers instead of lung features. This demonstrates the value of explainability methods. It also highlights the different goals of explainability methods and weakly supervised segmentation. Their goals are fundamentally different: weakly supervised segmentation aims to segment the pathology as precisely as possible, whereas explainability methods aim to reflect the neural network’s decision-making process as precisely as possible. Ideally, these two should align if the network does not rely on shortcuts–but this is not necessarily true, as shown^38,39^. Consequently, an evaluation based solely on the ability of a saliency method to localize a pathology may unintentionally favor a method that does not truly explain the network but highlights, for example, random spots in a specific body region over one that highlights the true decision rationale. Moreover, all localization metrics in this work are reported at the level of individual pathologies and aggregate lesions across the entire thorax; the available datasets do not contain standardized labels for apical, hilar, central, or basal regions, so we cannot stratify localization performance by anatomical subregion (Fig. 5).

Consider the following thought experiment: Imagine a neural network that achieves high accuracy in fracture detection on a hypothetical dataset. In this dataset, images with fractures consistently display a laterality marker “R,” whereas those without fractures have a marker “L.” Suppose the trained network relies entirely on these laterality markers for classification. Now, consider two explainability methods: Method *A* accurately highlights the true basis for classification. Method *B*, however, randomly highlights bone regions regardless of fracture presence. If evaluated solely by how often they correctly localize or identify the fracture, method *B* might appear superior to method *A*, as it occasionally overlaps with fractures by chance, whereas method *A* consistently highlights only laterality markers and never directly indicates fractures.

This underscores that evaluations relying solely on segmentation accuracy or hit rate are inadequate for comparing explainability methods. A concrete example is cardiomegaly: human annotators often contour or point to the entire enlarged cardiac silhouette, which is clinically natural, whereas saliency methods typically highlight only those edges of the heart or mediastinum that are most discriminative for the model. Both behaviours are reasonable from their respective perspectives, but they yield different spatial patterns and can therefore create apparent performance gaps between human and model explanations when compared against dense radiologist segmentations. As described by the authors of the CheXlocalize paper, they evaluate how well post-hoc saliency methods perform on localization tasks^25^. This, however, does not necessarily align with the goal of explaining the neural network’s decision as precisely as possible. The same holds true for evaluating saliency methods based on the visual assessment of saliency maps by human experts. Just because a saliency map appears to align with a human expert’s reasoning does not necessarily mean the network relied on the same visual features for classification. For a radiologist in the thought experiment, examining bones like method *B* would seem more intuitive when searching for a fracture than focusing on laterality markers like method *A*.

An effective explainability method must inherently reflect only the true underlying reasons behind the network’s classification decisions by design. In MedicalPatchNet, a key goal was to keep the model a drop-in replacement for a standard image-level classifier: it processes full images, relies solely on image-level labels, and does not use explicit masks or segmentation annotations to select regions. Interpretability is then achieved by restricting raw patch encodings to purely local information. Including all patches, even those at the image margins, is a deliberate design choice: it keeps the architecture fully annotation-free and allows the resulting saliency maps to expose potential shortcut features in peripheral regions (such as markers or border artifacts), rather than suppressing them a priori through manual masking. There is no communication between the information from different patches until the final averaging step. Therefore, if the network utilizes information from a patch for classification, the logits corresponding to that patch must directly reflect this. Combined with the drop-in design relative to the underlying backbone, this yields a model that can be used in place of a standard image-level classifier while making all contributing image regions explicitly visible. There is no alternative mechanism that allows such information to influence the final prediction unless it is explicitly represented in the generated saliency map.

Although several Multiple Instance Learning approaches^32–34^ similarly process images at the patch level and provide interpretability through learned attention mechanisms or transformer-based correlations, these methods have been primarily applied in histopathology. In contrast, MedicalPatchNet is specifically designed for chest X-ray analysis and deliberately avoids learnable pooling or inter-patch interactions. By averaging independently predicted patch logits, our method ensures that each patch’s influence on the final decision is direct and transparent, offering interpretability by design rather than relying on learned attribution weights.

### Limitations

Using our architecture comes with limitations. First, the reliance on only local information does not allow for classifications that depend on broader context, where information from spatially distant image regions must be aggregated–beyond the patch size. For example, pulmonary congestion is often associated both with an enlarged heart and with bilateral infiltrates. Such a condition requires integrating information across distant regions of the image, which cannot be captured by our patch-wise averaging strategy.

Second, MedicalPatchNet is primarily sensitive to local shortcut features that are confined to specific regions of the image (e.g. laterality markers, text overlays, medical devices, border artifacts). Dataset biases that act globally—such as vendor-specific post-processing or characteristic global contrast and texture patterns—will usually affect all patches similarly, so our saliency maps tend to appear relatively homogeneous and cannot meaningfully isolate the source of such global bias.

Third, the explainability we provide is restricted to spatial attribution at the patch level. Our saliency maps indicate where evidence for a class arises, but not which fine-grained visual concept within a patch is responsible; the internal feature representations of each patch-wise classifier therefore remain a black box and may require complementary concept-level analyses in future work.

Fourth, our quantitative localization analysis does not differentiate between anatomical subregions. Lesions in apical, perihilar, central, or basal lung regions are evaluated jointly for each pathology, because the available datasets do not provide structured region labels. As a result, potential differences in localization behaviour across specific anatomical areas are not captured by our current evaluation and should be investigated in future work once suitable region-level annotations become available.

### Conclusion

This study presents the new inherently explainable MedicalPatchNet architecture, which enables backtracking and decomposition of the exact contribution of each patch to the final classification. Unlike traditional post-hoc explainability methods that can produce misleading visualizations, MedicalPatchNet incorporates explainability directly into the model’s core architecture. The resulting patch-based saliency maps transparently reflect the exact contributions of individual image regions to each diagnostic decision. Empirical evaluations on the CheXpert and CheXlocalize datasets show that MedicalPatchNet not only achieves strong classification performance but also outperforms existing methods such as Grad-CAM and its variants in pathology localization accuracy when evaluated across true positive, false positive, and false negative cases. This demonstrates the feasibility of maintaining high diagnostic precision without sacrificing interpretability. MedicalPatchNet thus addresses a critical barrier to clinical AI adoption by delivering inherently reliable explanations that are accessible even to practitioners without extensive deep learning expertise. Future work could adapt this explainability approach to other settings, such as 3D imaging or multimodal classification, enabling quantification of the contribution of different modalities. In particular, the same patch-based architecture could be applied to volumetric patches or slabs in CT or MRI, or extended to multi-disease diagnostic tasks beyond chest X-rays, providing analogous self-explainable decompositions in more complex imaging scenarios. When applied to neural networks used in clinical settings, this approach may enhance trust, ultimately facilitating safer, more effective patient care through transparent AI-assisted diagnostics.

## Supplementary Information

Supplementary Information.

## Data Availability

The CheXpert dataset^41^ used for training is publicly available from the Stanford ML Group at https://stanfordmlgroup.github.io/competitions/chexpert/. The CheXlocalize dataset^25^, used for evaluation, is publicly available from the Stanford AIMI group at https://stanfordaimi.azurewebsites.net/datasets/23c56a0d-15de-405b-87c8-99c30138950c. The source code for our model, training, and evaluation, are publicly available on GitHub at github.com/TruhnLab/MedicalPatchNet. The model weights are publicly available on Hugging Face: https://huggingface.co/patrick-w/MedicalPatchNet
