# Supplementary material for: MedicalPatchNet: a patch-based self-explainable AI architecture for chest X-ray classification
Source: Sci Rep. 2026 Feb 20;16:7467. doi: 10.1038/s41598-026-40358-0 (PMC12929615; doi:10.1038/s41598-026-40358-0)
Supplement: Supplementary file 1 — Supplementary Information. [file 41598_2026_40358_MOESM1_ESM.pdf]

Supplementary Material for MedicalPatchNet: A Patch-Based Self-Explainable AI Architecture for Chest X-ray Classification

1 Mask Size Evaluation

To assess how lesion size affects localization performance, we stratified the CheXlocalize test set by ground-truth mask area and evaluated MedicalPatchNet separately for smaller and larger lesions. Specifically, for each pathology we split cases into those with a ground-truth segmentation mask area greater than the median and those with an area less than or equal to the median. Across all pathologies with available segmentations, the median fraction of image area covered by a ground-truth mask is 9.92%.

	Hit rate		mIoU	
Pathology	>Median	≤Median	>Median	≤Median
Lung Opacity	0.698	0.404	0.144	0.032
Atelectasis	0.746	0.364	0.133	0.017
Cardiomegaly	0.475	0.308	0.143	0.003
Consolidation	0.769	0.409	0.261	0.030
Edema	0.681	0.500	0.276	0.022
Enlarged Cardiomediastinum	0.423	0.167	0.177	0.002
Lung Lesion	1.000	0.231	0.206	0.005
Pleural Effusion	0.788	0.345	0.324	0.075
Pneumothorax	0.500	0.500	0.075	0.080
Support Devices	0.561	0.385	0.221	0.160

Table S1. Hit rate and mean Intersection over Union (mIoU) stratified by mask size. For each pathology, metrics are reported separately for lesions with ground-truth mask area greater than (">Median") or less than or equal to ("≤Median") the median lesion size.

When comparing the values in Table S1, a consistent pattern emerges: for almost all pathologies, hit rate and mIoU are markedly lower for lesions with ground-truth masks at or below the median size than for larger lesions. We attribute this effect to the fact that small lesions occupy only a few patches or pixels, making it more difficult for the most salient point to fall inside the ground-truth region and causing the IoU metric to penalize even minor spatial misalignment much more strongly than for larger, more extended pathologies.

2 Patch Size Analysis

To investigate the effect of patch size on classification performance, we evaluated MedicalPatchNet not only with a patch size of 64×64 pixels, which yields an 8×8 grid of patches per image, but also with patch sizes of 128×128 and 256×256 pixels, corresponding to 4×4 and 2×2 patches, respectively. Applying EfficientNetV2-S directly to the full image is equivalent to using a patch size of 512×512 pixels with a single patch, i.e. a 1×1 grid. As illustrated in Figure S1, the impact of patch size on AUROC is pathology-dependent. The most pronounced difference is observed for pneumonia: MedicalPatchNet with a patch size of 64×64 achieves an AUROC of 0.815, whereas the variant with a patch size of 256×256 reaches an AUROC of 0.906. In contrast, for some findings such as "Support Devices", the AUROC values for all tested patch sizes lie in a narrow range between 0.966 and 0.971. When comparing AUROC across patch sizes, as summarized in Figure S2, a general trend emerges in which larger patch sizes yield slightly higher average performance, even though all configurations operate within a relatively small AUROC range of approximately 0.900 to 0.911. It is important to emphasize, however, that increasing the patch size simultaneously reduces the spatial resolution of the saliency maps: once a patch containing a pathology is highlighted, a larger patch size leads to the corresponding heatmap covering a larger area of the image.

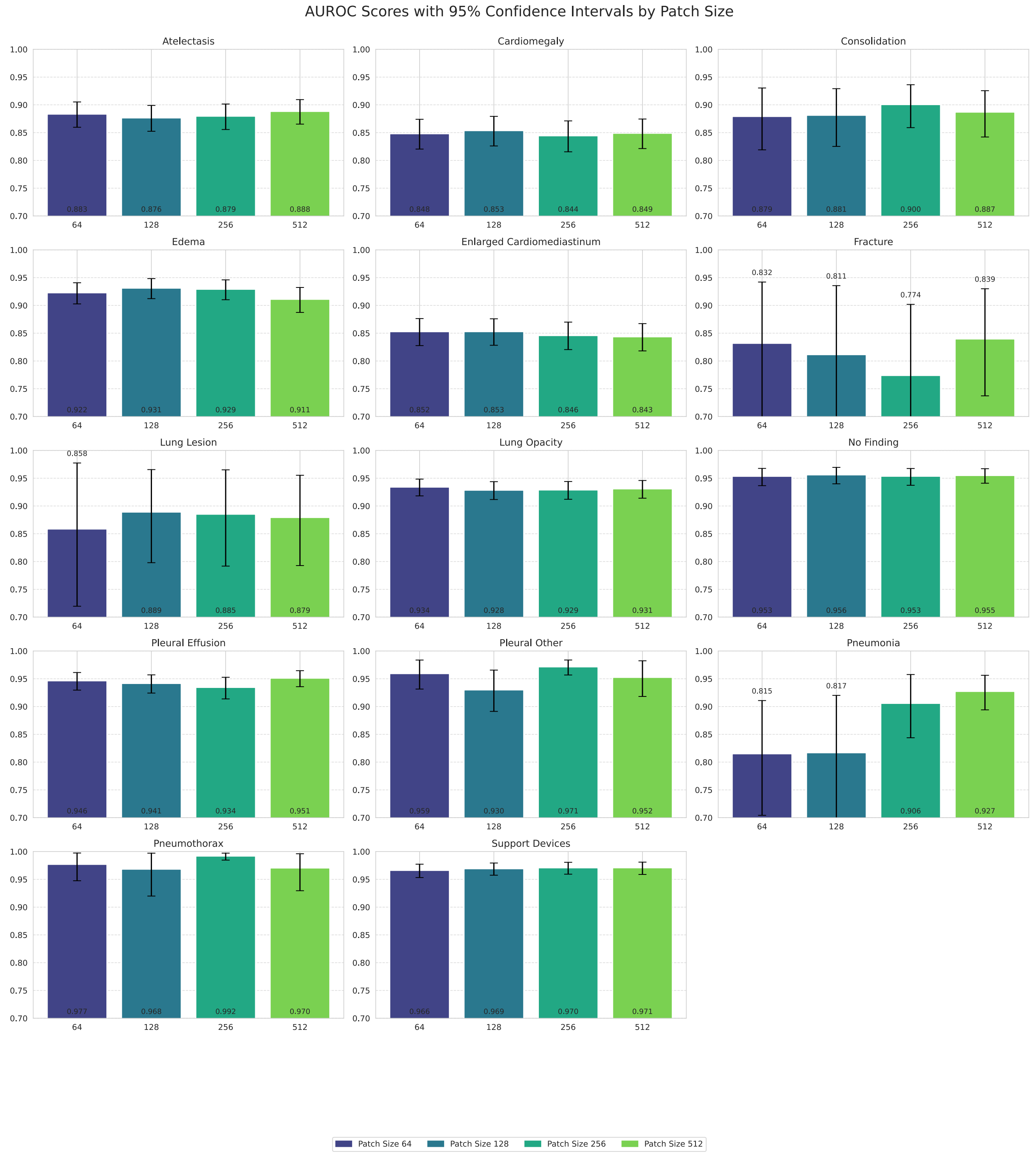

Figure S1. Classification performance (AUROC) of MedicalPatchNet for different patch sizes across all CheXpert pathologies. For each class, bars show the AUROC obtained with patch sizes of 64×64, 128×128, and 256×256 pixels, as well as the baseline EfficientNetV2-S model, which corresponds to processing the full 512×512 image as a single patch. Error bars indicate 95% confidence intervals.

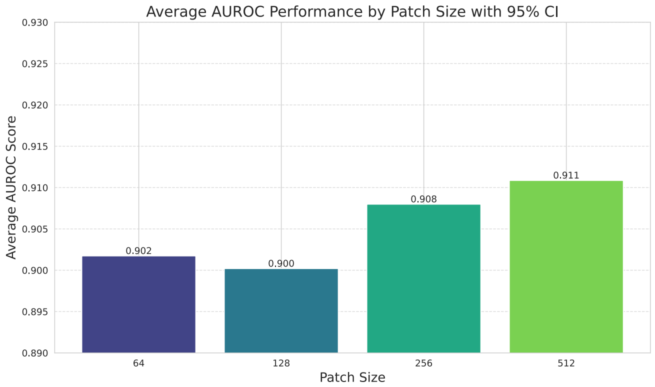

Figure S2. Average AUROC over all pathologies as a function of patch size. The four bars correspond to patch sizes of 64×64, 128×128, 256×256, and 512×512 pixels (the latter representing the standard EfficientNetV2-S image-level classifier). Error bars denote 95% confidence intervals.

3 Margin Analysis

MedicalPatchNet was deliberately designed as a drop-in replacement for conventional image-level classifiers (such as ResNet, EfficientNet, or Vision Transformers). Consequently, it operates directly on the full radiograph without requiring any additional inputs, for example lung masks that would restrict the analysis to predefined regions of interest. This raises the question to what extent patches at the image margins, which are often clinically less relevant, contribute to the final predictions. To investigate this, we analysed how strongly individual patches influence the output by computing the average absolute patch logits over the CheXpert test set. Figure S3 shows these mean absolute logit magnitudes, first aggregated over all classes and then separately for each CheXpert label. As visible from these maps, the patches that most strongly influence the classification form a lung-shaped region in the central part of the image, whereas patches at the margins generally contribute much less.

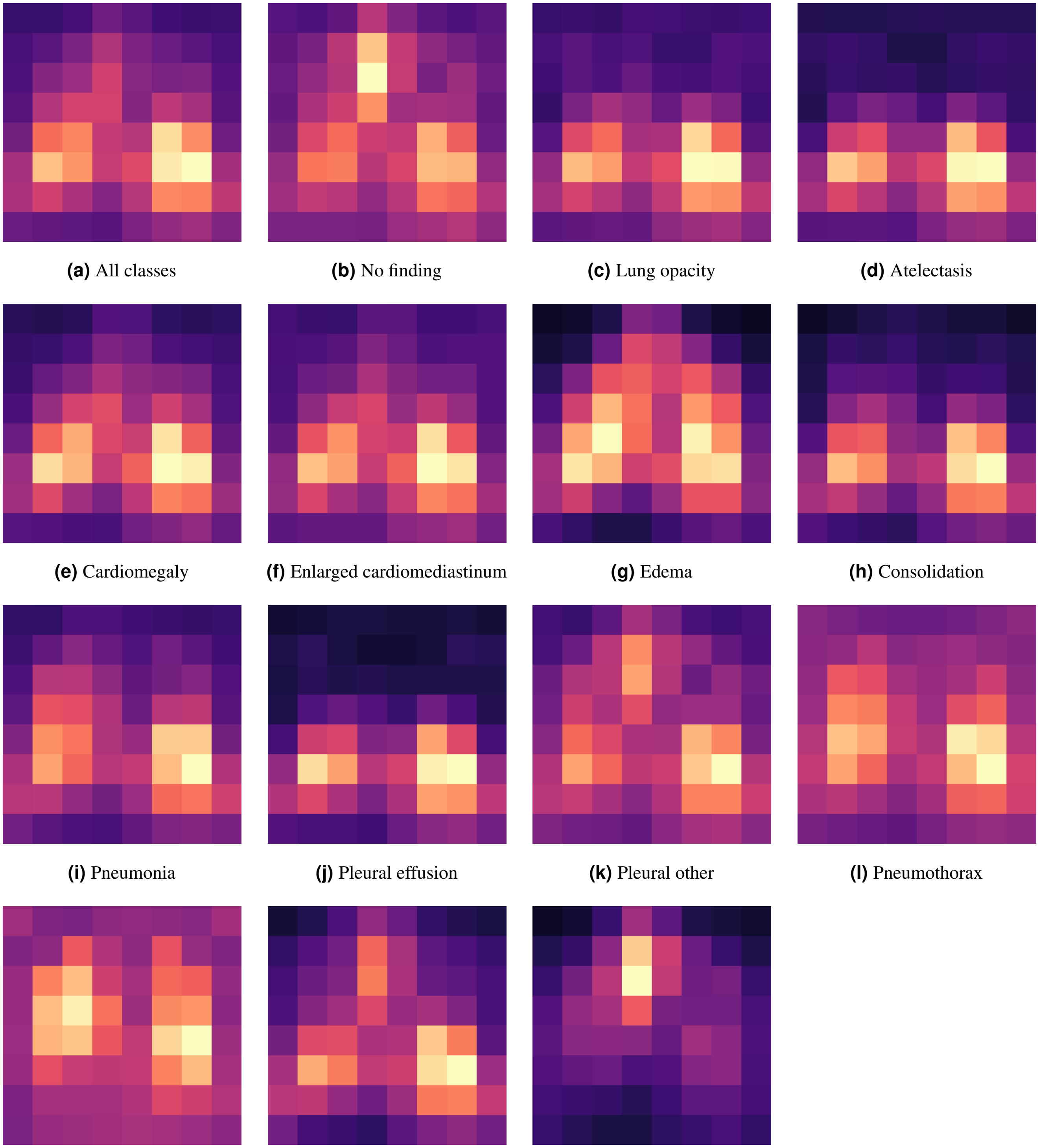

(a) All classes
(b) No finding
(c) Lung opacity
(d) Atelectasis
(e) Cardiomegaly
(f) Enlarged cardiomediastinum
(g) Edema
(h) Consolidation
(i) Pneumonia
(j) Pleural effusion
(k) Pleural other
(l) Pneumothorax
(m) Lung lesion
(n) Fracture
(o) Support devices

Figure S3. Patch-wise average absolute logit magnitude for a single decoding step, averaged over the CheXpert test set. The first map shows the mean over all classes; the remaining maps correspond to individual CheXpert labels (the Airspace opacity label is reported as Lung opacity).

4 Evaluation on natural images

To assess how well our architecture transfers to non-medical imagery, we additionally evaluated it on multi-label object classification using the PASCAL VOC 2012 dataset[1]. We used the official validation split for testing and randomly divided the official training split into 80% training and 20% validation images. Each image was assigned all object classes that were present in its annotations.

For this experiment, we trained MedicalPatchNet with the same optimization and augmentation settings as in the main paper. Input images were zero-padded to a square canvas, resized to 512×512 pixels, and subdivided into a grid of 8×8 non-overlapping patches of size 64×64 pixels. As backbone, we again used EfficientNetV2-S.

We compared MedicalPatchNet to a standard EfficientNetV2-S baseline that processes the full image in a single pass and therefore has access to the complete global context. Both models were trained under identical conditions, differing only in their patch-based versus image-level architectures. The resulting performance metrics on the VOC 2012 validation set for different training durations are summarized in Table S2.

	10 epochs		30 epochs		100 epochs	
	MedPatchNet	EffNetV2-S	MedPatchNet	EffNetV2-S	MedPatchNet	EffNetV2-S
AUROC	0.915	0.980	0.959	0.974	0.951	0.972
Accuracy	0.945	0.979	0.966	0.977	0.965	0.977
Sensitivity	0.294	0.822	0.640	0.814	0.644	0.820
Specificity	0.988	0.990	0.989	0.990	0.988	0.989
Precision	0.640	0.873	0.822	0.853	0.823	0.849
F1-Score	0.368	0.845	0.717	0.831	0.721	0.834

Table S2. Classification performance on the PASCAL VOC 2012 validation set for MedicalPatchNet ("MedPatchNet") and a standard EfficientNetV2-S classifier ("EffNetV2-S") after 10, 30, and 100 training epochs. All metrics are averaged over classes in the multi-label setting.

Compared to the chest X-ray experiments, the AUROC gap between MedicalPatchNet and EfficientNetV2-S on natural images is slightly larger: after 30 epochs, EfficientNetV2-S outperforms MedicalPatchNet by an absolute AUROC margin of 0.015. Nonetheless, MedicalPatchNet reaches competitive performance while retaining its inherent patch-wise explainability.

	MedicalPatchNet		EfficientNetV2-S		
Pathology	Scaled Encodings	Raw Encodings	Grad-CAM	Grad-CAM++	Eigen-CAM
Lung Opacity	0.040 [0.036–0.044]	0.040 [0.036–0.044]	0.097 [0.087–0.108]	**0.101** [0.091–0.112]	<u>0.101</u> [0.090–0.111]
Atelectasis	0.023 [0.019–0.026]	0.023 [0.019–0.026]	<u>0.057</u> [0.049–0.066]	**0.061** [0.052–0.070]	0.057 [0.049–0.066]
Cardiomegaly	0.036 [0.032–0.041]	0.036 [0.032–0.041]	<u>0.061</u> [0.053–0.069]	**0.063** [0.055–0.071]	0.060 [0.052–0.068]
Consolidation	**0.044** [0.030–0.059]	<u>0.015</u> [0.010–0.021]	0.012 [0.008–0.017]	0.012 [0.008–0.017]	0.012 [0.008–0.017]
Edema	**0.106** [0.086–0.129]	0.048 [0.039–0.059]	0.047 [0.038–0.057]	<u>0.050</u> [0.040–0.060]	0.048 [0.039–0.058]
Enlarged Card.	0.078 [0.071–0.085]	0.078 [0.071–0.085]	0.129 [0.117–0.140]	**0.136** [0.123–0.148]	<u>0.133</u> [0.121–0.145]
Lung Lesion	<u>0.006</u> [0.002–0.010]	**0.029** [0.011–0.050]	0.003 [0.001–0.005]	0.003 [0.001–0.006]	0.003 [0.001–0.006]
Pleural Effusion	**0.109** [0.088–0.132]	<u>0.061</u> [0.049–0.074]	0.031 [0.024–0.039]	0.033 [0.025–0.041]	0.026 [0.020–0.032]
Pneumothorax	**0.079** [0.030–0.135]	<u>0.060</u> [0.019–0.114]	0.004 [0.001–0.006]	0.004 [0.001–0.006]	0.002 [0.001–0.004]
Support Devices	<u>0.166</u> [0.156–0.176]	**0.171** [0.158–0.184]	0.075 [0.068–0.083]	0.075 [0.068–0.082]	0.047 [0.041–0.052]
Mean	**0.069**	<u>0.056</u>	0.052	0.054	0.049

Table S3. Mean Intersection over Union (mIoU) for all cases (true positive, false positive, and false negative). The highest and second-highest means per row are bold and underlined, respectively. Brackets denote 95% confidence intervals.

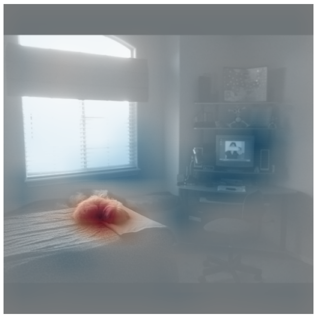

(a) Dog

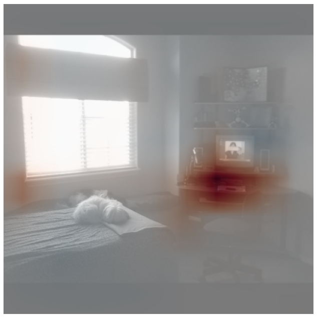

(b) TV-Monitor

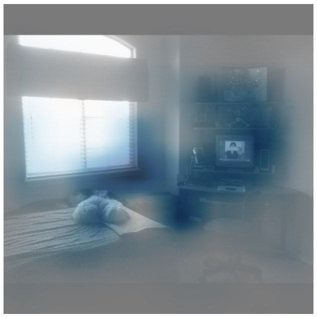

(c) Aeroplane

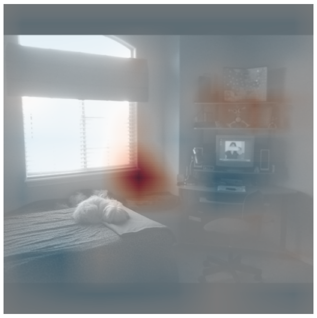

(d) Potted Plant

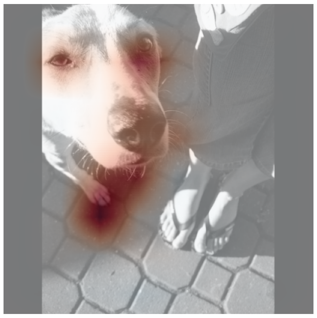

(e) Dog

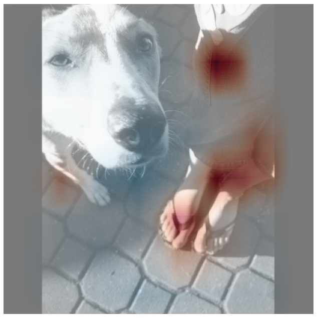

(f) Person

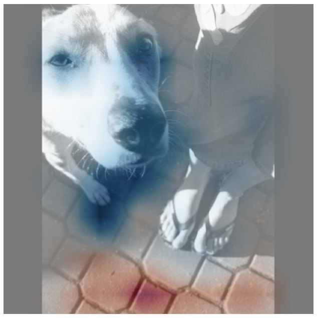

(g) Bird

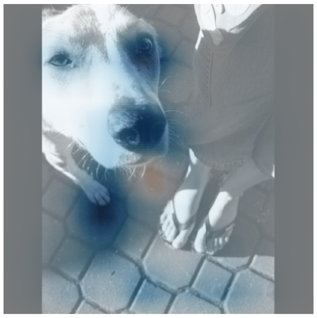

(h) Sheep

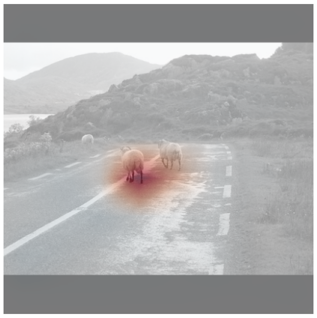

(i) Sheep

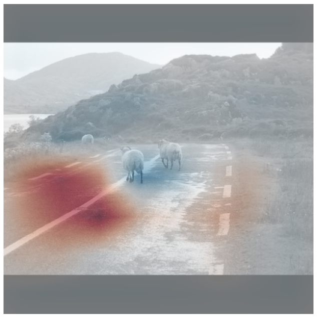

(j) Car

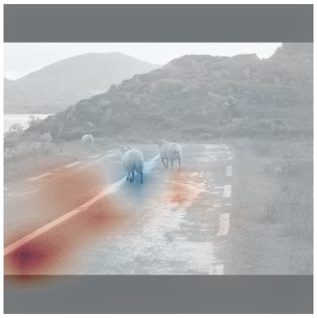

(k) Bicycle

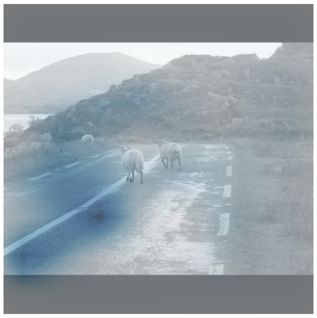

(l) Sofa

Figure S4. Representative VOC 2012 examples with corresponding saliency maps produced by MedicalPatchNet.

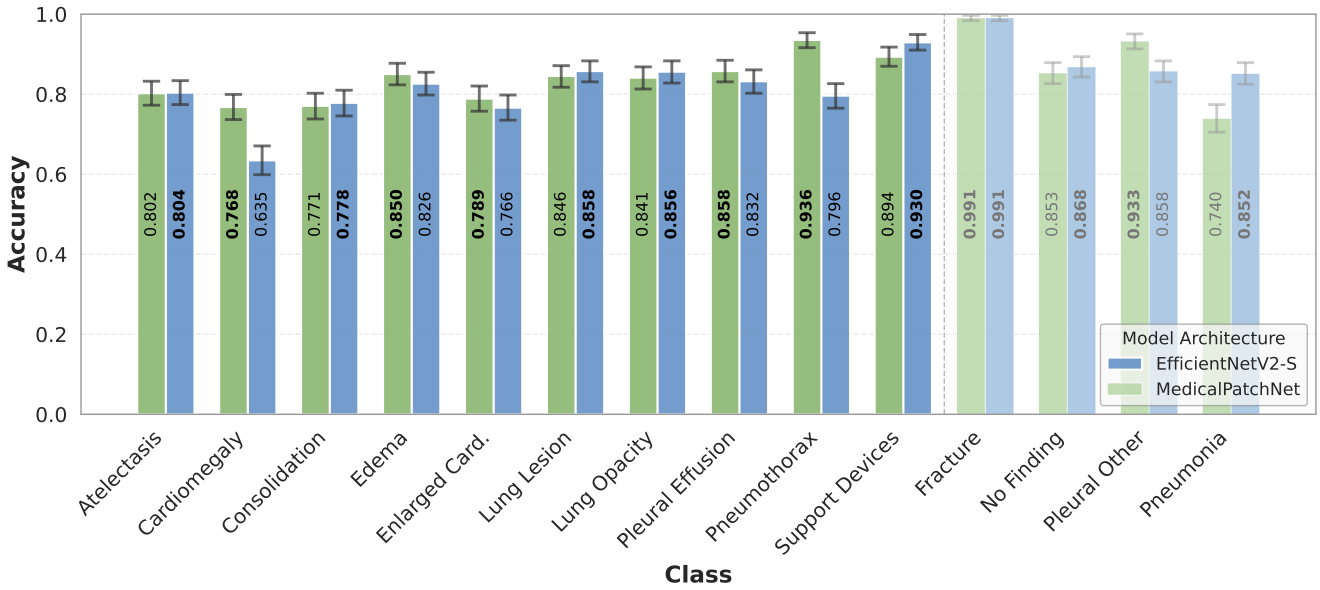

Figure S5. Comparison of accuracy between MedicalPatchNet and EfficientNetV2-S. The classification threshold for each class was determined by maximizing the sum of sensitivity and specificity on the validation set. Error bars indicate 95% confidence intervals.

	MedicalPatchNet		EfficientNetV2-S		
Pathology	Scaled Encodings	Raw Encodings	Grad-CAM	Grad-CAM++	Eigen-CAM
Lung Opacity	0.087 [0.081–0.092]	0.087 [0.082–0.092]	0.210 [0.197–0.223]	**0.219** [0.205–0.232]	<u>0.218</u> [0.204–0.232]
Atelectasis	0.086 [0.079–0.092]	0.086 [0.079–0.092]	0.213 [0.195–0.232]	**0.228** [0.210–0.247]	<u>0.216</u> [0.197–0.234]
Cardiomegaly	0.139 [0.135–0.144]	0.138 [0.134–0.143]	<u>0.232</u> [0.220–0.244]	**0.241** [0.229–0.252]	0.228 [0.217–0.239]
Consolidation	**0.255** [0.217–0.295]	0.232 [0.196–0.268]	0.237 [0.195–0.282]	<u>0.238</u> [0.195–0.284]	0.233 [0.186–0.280]
Edema	0.296 [0.265–0.323]	0.288 [0.262–0.312]	0.377 [0.350–0.402]	**0.403** [0.377–0.426]	<u>0.389</u> [0.364–0.412]
Enlarged Card.	0.175 [0.171–0.180]	0.175 [0.171–0.180]	0.289 [0.279–0.300]	**0.305** [0.295–0.315]	<u>0.299</u> [0.289–0.310]
Lung Lesion	0.071 [0.038–0.116]	0.109 [0.065–0.162]	0.146 [0.082–0.206]	**0.171** [0.087–0.259]	<u>0.164</u> [0.072–0.251]
Pleural Effusion	**0.221** [0.193–0.248]	0.069 [0.048–0.092]	0.174 [0.148–0.201]	<u>0.183</u> [0.156–0.210]	0.145 [0.121–0.169]
Pneumothorax	0.167 [0.085–0.263]	**0.235** [0.153–0.322]	<u>0.232</u> [0.156–0.311]	0.231 [0.145–0.322]	0.158 [0.083–0.234]
Support Devices	<u>0.186</u> [0.177–0.196]	**0.218** [0.207–0.228]	0.160 [0.151–0.169]	0.159 [0.149–0.168]	0.099 [0.091–0.106]
Mean	0.168	0.164	<u>0.227</u>	**0.238**	0.215

Table S4. Mean Intersection over Union (mIoU) evaluated over the true positive cases only. The highest and second-highest means per row are bold and underlined, respectively. Brackets denote 95% confidence intervals.

5 Further Examples

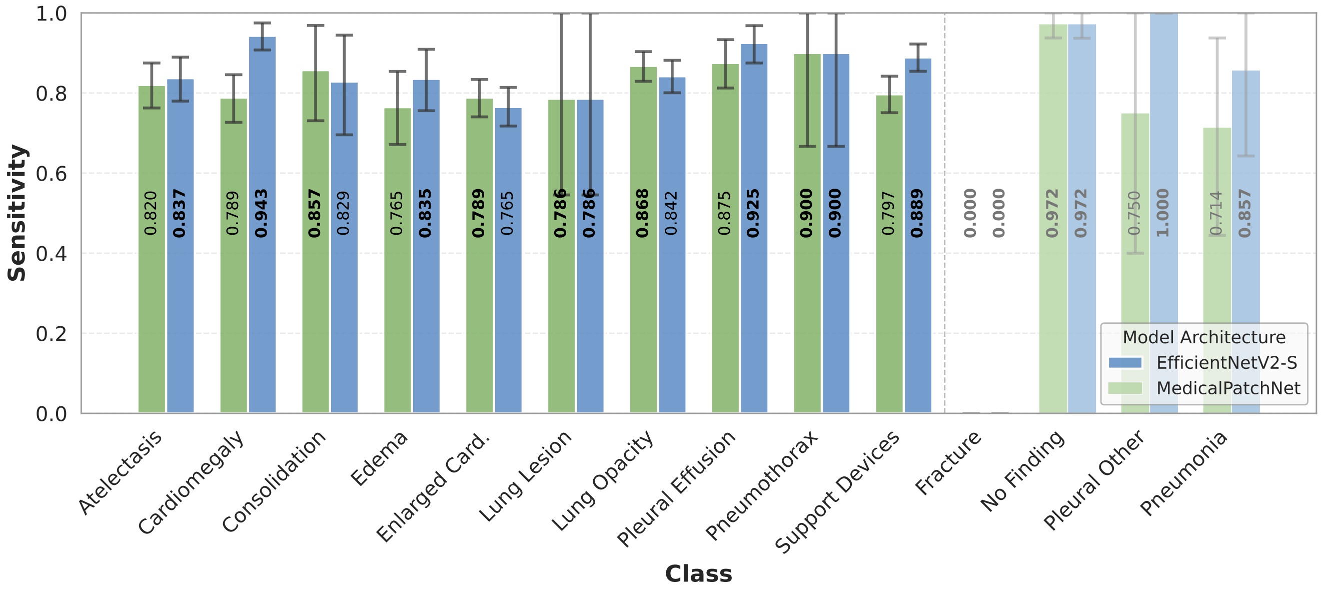

Figure S6. Comparison of sensitivity between MedicalPatchNet and EfficientNetV2-S. The classification threshold for each class was determined by maximizing the sum of sensitivity and specificity on the validation set. Error bars indicate 95% confidence intervals.

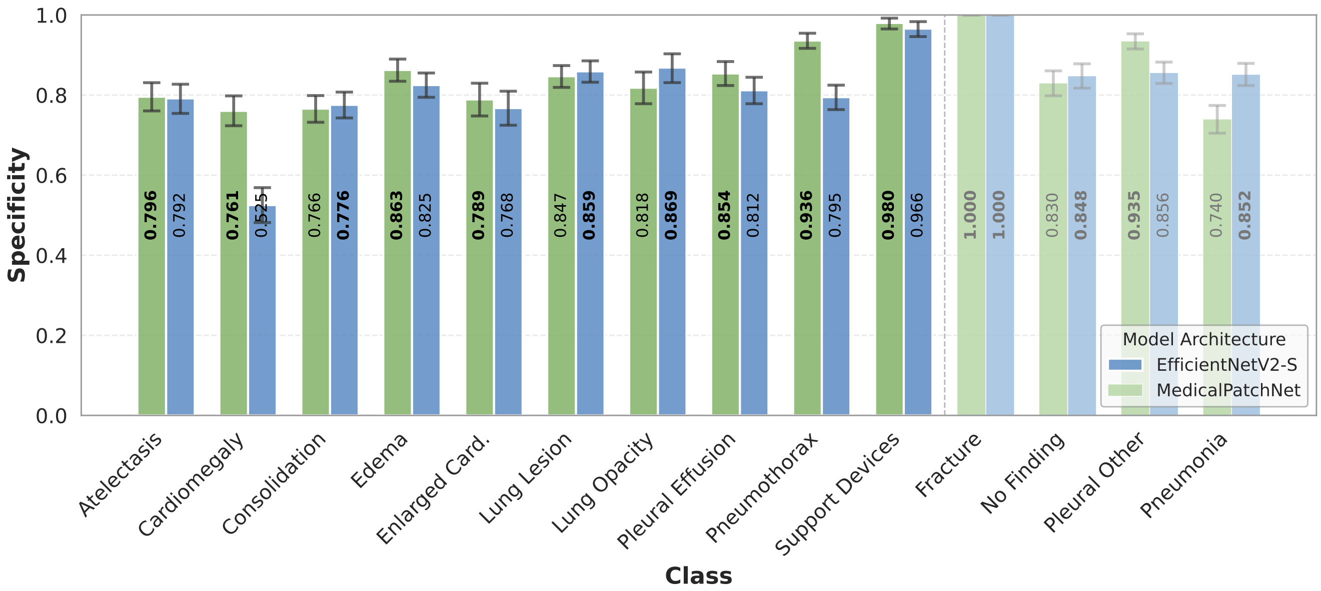

Figure S7. Comparison of specificity between MedicalPatchNet and EfficientNetV2-S. The classification threshold for each class was determined by maximizing the sum of sensitivity and specificity on the validation set. Error bars indicate 95% confidence intervals.

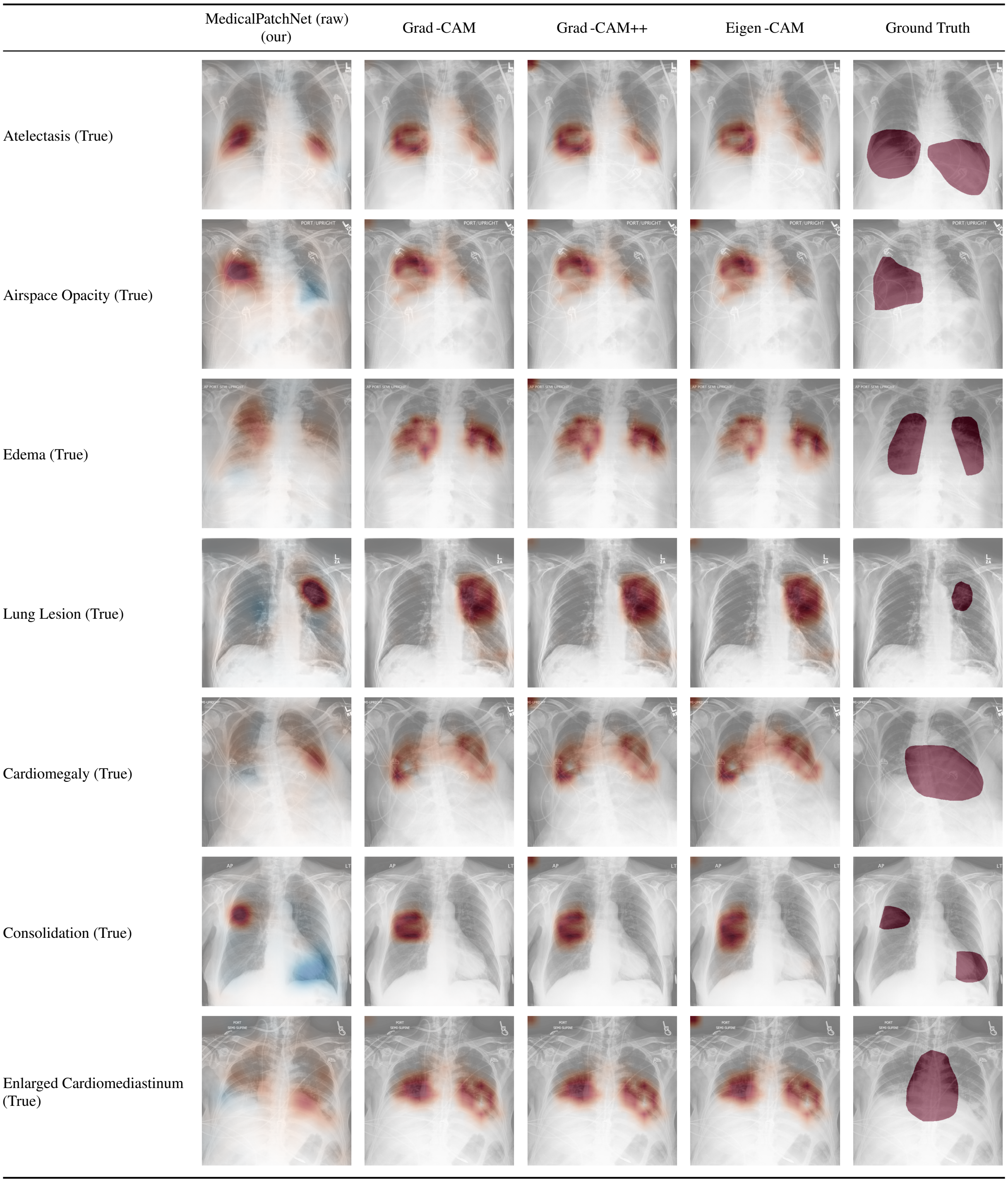

Figure S8. Representative saliency maps produced by MedicalPatchNet and three post-hoc methods. Each row displays the same chest X-ray for a given pathology together with its ground-truth label ("True" or "False"). Columns compare MedicalPatchNet's raw patch logits with Grad-CAM, Grad-CAM++, and Eigen-CAM applied to an EfficientNetV2-S baseline. In the MedicalPatchNet maps, red denotes evidence supporting the class and blue denotes evidence against it, whereas Grad-CAM–based maps visualize only positive (red) contributions. Eigen-CAM is class-agnostic and therefore does not generate class-specific saliency maps. Interestingly, for the wrongly diagnosed pneumothorax, all four explainability methods point to the chest tube, revealing that the model used a shortcut, with MedicalPatchnet denoting its course most clearly.

Performance Difference Explanation

Our results differ from those in the original CheXlocalize paper[2] because of methodological choices, not inherent limitations of our approach: we trained and evaluated single models—one MedicalPatchNet and one EfficientNetV2-S—whereas Saporta et al. generated 120 checkpoints for each of three models, chose the ten best checkpoints per pathology, and ensembled those selections, a strategy that inflates metrics relative to single-model evaluation. The preprint version of their work[1] reports single-network results comparable to ours, reinforcing that the observed gap stems from ensemble versus single-model evaluation.

6 Self-Explainable Methods

As our method falls in the category of self explainable methods, its natural to compare it to other self explainable approaches. For this we compare it to the ProtoPNet[3] and the PIPNet[4]. We haven't made a comparison to the XProtoNet[5], as the code was not publicly available to reproduce it.

ProtoPNet

ProtoPNet[3] is a self-explainable architecture that classifies images by comparing internal image representations to a small set of learned prototypes. Each prototype is intended to represent a recurring visual pattern, and predictions are obtained by aggregating evidence of the form "this region looks like that prototype". In contrast to post-hoc saliency methods, the explanation is part of the forward pass: the model decision is explicitly constructed from prototype matches.

Prototype matching in latent space

At a high level, ProtoPNet consists of (i) a convolutional backbone that maps an input image x to a spatial feature map $z = f(x)$, (ii) a set of learnable prototypes $\{p_j\}$ that live in the same latent space as local feature vectors of z, and (iii) a linear classification layer on top of prototype similarity scores.

Concretely, each prototype p_j is compared to all spatial locations of the feature map z by computing a distance (typically an ℓ_2 distance in feature space) between p_j and each local feature vector of z. This yields a spatial distance map per prototype, which is converted into a similarity map. ProtoPNet then performs a spatial max operation per prototype, so that each prototype contributes a single scalar similarity score given by its best-matching location in the image. The final class logits are computed as a weighted sum of these prototype scores. In our multi-label setting, the resulting logits are passed through a sigmoid to obtain per-label probabilities.

Push / projection step (prototype grounding)

A key ingredient for interpretability is the prototype grounding procedure (often called push or projection). After (or during) training, each prototype is replaced by the latent feature vector of the closest training patch (in the backbone feature space), typically constrained to come from an image that contains the prototype's target class. This step ties each abstract prototype vector to an actual, human-inspectable training image region: the model can now display, for each prototype, the training patch it was projected onto and the corresponding most similar patch in a test image. Importantly, this grounding does not change the inference rule (matching in latent space + max aggregation + linear classifier); it only enforces that the stored prototypes correspond to real training patches, making the "this looks like that" explanations visually meaningful.

Results on CheXlocalize

Following the reviewer suggestion, we evaluated ProtoPNet on the CheXlocalize test set (668 samples, 14 labels). Overall performance was close to random, with a macro-average AUROC of 0.502. Notably, threshold selection degenerated for most labels: the optimal threshold was 0.0 for 13/14 labels, which results in predicting nearly all cases as positive (sensitivity ≈ 1.0 and specificity ≈ 0.0), consistent with poor separability of the predicted scores.

[1] https://www.medrxiv.org/content/10.1101/2021.02.28.21252634v1

Label	AUROC	Accuracy	Sensitivity	Specificity	F1
No Finding	0.523	0.163	1.000	0.000	0.281
Enlarged Cardiomediastinum	0.507	0.446	1.000	0.000	0.617
Cardiomegaly	0.521	0.262	1.000	0.000	0.415
Lung Opacity	0.479	0.464	1.000	0.000	0.634
Lung Lesion	0.490	0.021	1.000	0.000	0.041
Edema	0.512	0.127	1.000	0.000	0.226
Consolidation	0.525	0.052	1.000	0.000	0.100
Pneumonia	0.492	0.021	1.000	0.000	0.041
Atelectasis	0.493	0.266	1.000	0.000	0.421
Pneumothorax	0.493	0.015	1.000	0.000	0.030
Pleural Effusion	0.520	0.180	1.000	0.000	0.305
Pleural Other	0.493	0.012	1.000	0.000	0.024
Fracture	0.497	0.991	0.000	1.000	0.000
Support Devices	0.489	0.472	1.000	0.000	0.641
Average	0.502	0.249	0.929	0.071	0.270

Table S5. ProtoPNet performance on the CheXlocalize test set (14-label multi-label classification). Metrics are reported per label and averaged across labels.

Why ProtoPNet underperformed

We hypothesize that the poor performance is primarily caused by a mismatch between ProtoPNet's training objective and the properties of chest X-ray multi-label classification.

First, ProtoPNet was originally designed for single-label classification, where each image belongs to exactly one class. Its prototype assignment and separation mechanisms implicitly assume that evidence should be class-exclusive: patches that support class A are simultaneously treated as evidence against class B. In chest X-rays, however, labels frequently co-occur (e.g., cardiomegaly with edema), so the same image region can legitimately support multiple labels. In such a setting, enforcing strong separation between prototypes of different classes can become contradictory and may push the representation toward trivial solutions.

Second, ProtoPNet aggregates prototype evidence via a spatial max over the feature map. This makes the prediction highly sensitive to a single best-matching location per prototype. For radiographs, many findings are subtle, extended, or context-dependent (e.g., cardiomegaly depends on global thoracic context rather than a single local texture). A max-based evidence model can therefore be brittle: it may either lock onto ubiquitous, non-specific structures (ribs, mediastinum edges, text overlays, devices) or produce near-constant similarity scores that offer little discriminative power, both of which are consistent with AUROCs close to 0.5.

Third, strong class imbalance (especially for rare labels such as fracture) interacts poorly with a fixed prototype budget per class. If prototypes cannot reliably ground onto truly class-specific patches during projection, they may collapse onto background patterns; the subsequent linear layer can then drift into degenerate threshold behaviour (here: near-always-positive predictions for most labels and near-always-negative for fracture), yielding high sensitivity but vanishing specificity.

Overall, these observations suggest that ProtoPNet, in its standard form, is not well aligned with the multi-label, co-occurring, and often spatially diffuse nature of chest X-ray findings. Addressing this likely requires multi-label-aware prototype objectives (e.g., relaxing inter-class separation for co-occurring labels), alternative evidence pooling (beyond a pure max), and/or prototype allocation strategies that reflect the prevalence and heterogeneity of each pathology.

PIPNet

Method overview

PIP-Net[4] (Patch-based Intuitive Prototypes Network) is a prototype-based, inherently interpretable classifier that aims to explain predictions via a set of prototypical visual patterns. Conceptually, the model acts like a "scoring sheet": it measures how strongly an input image matches each learned prototype and then aggregates these prototype scores with non-negative class weights. The non-negativity constraint is intended to support case-based reasoning, where prototypes can only add evidence for a class (rather than subtracting it).

Prototype scoring and aggregation

PIPNet turns an image into a set of prototype activations and then combines them into class scores in a strictly additive way. Conceptually, the backbone produces a spatial feature map with K channels; PIPNet treats each channel as one prototype, so in

our configuration ($K = 768$ with a ConvNeXt-Tiny backbone) the model has 768 prototypes.

At each spatial location, the model computes how strongly each prototype "matches" the local image content, and then normalizes these responses across the K prototypes (so prototypes compete for attention at each location). For each prototype k, PIPNet keeps only its strongest match anywhere in the image, yielding one scalar score $s_k(x)$ per prototype (intuitively: "how much does the image contain something that looks like prototype k?").

A final non-negative linear layer then aggregates these prototype scores into one score per class by a weighted sum,

$$\ell_c(x) = \sum_{k=1}^{K} w_{c,k}\, s_k(x), \qquad w_{c,k} \geq 0,$$

so prototypes can only contribute positive evidence for a class. Explanations follow directly: a class is supported by those prototypes with large contributions $w_{c,k}s_k(x)$, and each such prototype can be localized by the image region where its maximum activation was attained.

Training recipe used in our experiments

We used the authors' public reference implementation from their GitHub repository (github.com/M-Nauta/PIPNet) and applied it to the CheXpert dataset. In this implementation, training follows a two-stage procedure.

In the first stage, the network is trained without using class labels to shape the prototype space. The objective encourages prototype activations to be stable under image augmentations (i.e., two augmented views of the same image should activate similar prototypes) and includes an additional regularization term that discourages overly sparse prototype usage. During this stage, the final classification layer is kept fixed (not updated), so the model focuses on learning a prototype representation rather than class mappings.

In the second stage, the model is fine-tuned with supervision to learn how prototypes map to target classes via the non-negative classification layer. The implementation enforces sparsity in this layer by repeatedly shrinking small weights toward zero during training, which promotes a small set of "active" prototypes per class. At inference time, weak prototype activations are additionally thresholded (set to zero), so only sufficiently strong prototype matches contribute to the final class scores. As a result, the overall decision is intentionally driven by a limited number of high-confidence prototype matches, which is meant to make explanations concise and prototype-based.

Results on chest X-ray classification

We evaluated PIPNet on the CheXpert-derived test set used throughout this work (668 images, 14 labels; the same image subset as the CheXlocalize test split, but evaluated for classification only). A critical failure mode emerged: the trained model produced meaningful predictions for only two labels, while all other labels collapsed to degenerate outputs (Table S6). Concretely, No Finding achieved an AUROC of 0.8374 and Support Devices reached an AUROC of 0.6127, whereas the remaining 12 labels yielded zero scores across all reported metrics in our evaluation.

Label	AUROC	Accuracy	Sensitivity	Specificity	F1
No Finding	0.8374	0.8323	0.7982	0.8390	0.6084
Enlarged Cardiomediastinum	0.0000	0.0000	0.0000	0.0000	0.0000
Cardiomegaly	0.0000	0.0000	0.0000	0.0000	0.0000
Lung Opacity	0.0000	0.0000	0.0000	0.0000	0.0000
Lung Lesion	0.0000	0.0000	0.0000	0.0000	0.0000
Edema	0.0000	0.0000	0.0000	0.0000	0.0000
Consolidation	0.0000	0.0000	0.0000	0.0000	0.0000
Pneumonia	0.0000	0.0000	0.0000	0.0000	0.0000
Atelectasis	0.0000	0.0000	0.0000	0.0000	0.0000
Pneumothorax	0.0000	0.0000	0.0000	0.0000	0.0000
Pleural Effusion	0.0000	0.0000	0.0000	0.0000	0.0000
Pleural Other	0.0000	0.0000	0.0000	0.0000	0.0000
Fracture	0.0000	0.0000	0.0000	0.0000	0.0000
Support Devices	0.6127	0.6332	0.2349	0.9887	0.3766
Average	0.1036	0.1047	0.0738	0.1305	0.0704

Table S6. PIPNet performance on the CheXpert-derived test set (668 images, 14 labels). Values are point estimates (no bootstrap confidence intervals available for this experiment).

Why might PIPNet fail here?

The observed collapse is most plausibly explained by a combination of interacting factors. Importantly, the following are hypotheses inferred from the observed behavior and from the training configuration; confirming them would require targeted ablations.

First, we hypothesize a mismatch between the default PIPNet learning objective and the CheXpert setting. CheXpert is genuinely multi-label, with frequent co-occurrence of findings. In contrast, the training recipe we used enforces competition between classes, which effectively encourages a single "winning" label per image. In such a regime, gradients may be systematically biased toward frequent or visually dominant labels, while suppressing learning signals for concurrent positives. This could explain why the model appears to concentrate on a very small subset of labels and fails to develop usable decision rules for most findings.

Second, we hypothesize that the combination of (i) a non-negative classifier, (ii) explicit sparsity enforcement during training, and (iii) inference-time thresholding of weak prototype activations can lead to premature collapse. If small class-to-prototype weights are repeatedly driven to exactly zero early in training, many labels may lose most of their effective supervised pathways. Once a label has effectively "lost access" to prototypes, recovering becomes difficult because its gradients cannot easily re-establish informative prototype-to-class connections under the same sparsifying dynamics.

Third, we hypothesize that the self-supervised pretraining objective can dominate representation learning in this medical domain. Prototype channels may become highly invariant to augmentations (as intended) but not sufficiently aligned with subtle radiographic cues required for pathology discrimination. The supervised stage would then need to re-purpose these prototypes for clinical findings while simultaneously operating under strong sparsity pressure, which is an unfavorable optimization problem.

Fourth, extreme class imbalance likely exacerbates all of the above. Several findings are rare in the evaluated split (e.g., pneumothorax, fracture, pleural other), and in the absence of explicit imbalance-aware weighting, these labels contribute comparatively little to the loss. We hypothesize that this makes them particularly prone to being ignored during optimization, further increasing the probability of degenerate outputs.

Finally, some thoracic findings are spatially diffuse or context-dependent (e.g., global opacity patterns or shape changes spanning large anatomical regions). We hypothesize that capturing such signals via a small number of local max-pooled prototype matches may be intrinsically difficult, which could further bias the model toward trivial solutions focused on a few visually distinct categories.

Overall, in the present configuration, PIPNet did not provide a viable performance–interpretability trade-off for chest X-ray multi-label classification: for most labels, the model failed to learn usable predictors, and consequently prototype-based explanations would not be meaningful in those cases.

Why we did not perform an extensive CheXlocalize evaluation for ProtoPNet and PIPNet

In principle, CheXlocalize would be the natural benchmark to compare localization behavior across self-explainable methods. However, localization metrics on CheXlocalize are only meaningful if the underlying classifier has learned a non-trivial decision function for the target labels; otherwise, saliency maps (and any thresholded masks derived from them) largely reflect noise or degenerate prediction behavior rather than clinically interpretable evidence.

In our experiments, both ProtoPNet and PIPNet were trained on CheXpert using the authors' public implementations and their default training hyperparameters. Under this setting, neither method produced a reliable multi-label chest X-ray classifier: ProtoPNet remained close to chance level (macro AUROC ≈ 0.5) with largely degenerate threshold behavior, and PIPNet collapsed to meaningful predictions for only two labels while yielding trivial outputs for the remaining classes. Given these failure modes at the classification level, a full CheXlocalize localization analysis would not provide informative or fair conclusions about explainability, because the resulting maps would primarily quantify artifacts of collapse rather than genuine model reasoning.

7 Generalizability across backbones (ConvNeXt)

To assess whether MedicalPatchNet's patch-wise, self-explainable aggregation generalizes beyond the EfficientNet family, we repeated the CheXpert/CheXlocalize classification evaluation using a ConvNeXt-Base backbone. We trained (i) *MedicalPatchNet – ConvNextBase* and (ii) a standard *ConvNext – 512×512* image-level classifier under identical preprocessing and optimization settings. Table S7 reports mean AUROC, accuracy, sensitivity, and specificity averaged across the 10 CheXlocalize labels (*) and across all 14 CheXpert labels (All). For the 10-label average (*), *Fracture, No Finding, Pleural Other, and Pneumonia* are excluded.

Model	AUROC		Accuracy		Sensitivity		Specificity	
	*	All	*	All	*	All	*	All
MedicalPatchNet – ConvNextBase	0.903	0.898	0.853	0.863	0.737	0.719	0.862	0.869
ConvNext – 512×512	0.903	0.899	0.857	0.865	0.755	0.737	0.867	0.871
Difference	0.000	-0.001	-0.004	-0.002	-0.018	-0.018	-0.005	-0.002

Table S7. Classification performance comparison using a ConvNeXt backbone. The metrics represent average values computed across the 10 classes from the CheXlocalize dataset (*) and across all 14 classes from the CheXpert dataset (All). For the 10-class average (*), Fracture, No Finding, Pleural Other, and Pneumonia were excluded.

References

1. Everingham, M. & Winn, J. The pascal visual object classes challenge 2012 (voc2012) development kit. *Pattern Analysis, Stat. Model. Comput. Learn. Tech. Rep* **8**, 2–5 (2011).

2. Saporta, A. *et al.* Benchmarking saliency methods for chest x-ray interpretation. *Nat. Mac. Intell.* **4**, 867–878, DOI: 10.1038/S42256-022-00536-X (2022).

3. Chen, C. *et al.* This looks like that: Deep learning for interpretable image recognition. In Wallach, H. M. *et al.* (eds.) *Advances in Neural Information Processing Systems 32: Annual Conference on Neural Information Processing Systems 2019, NeurIPS 2019, December 8-14, 2019, Vancouver, BC, Canada*, 8928–8939 (2019).

4. Nauta, M., Schlötterer, J., van Keulen, M. & Seifert, C. Pip-net: Patch-based intuitive prototypes for interpretable image classification. In *IEEE/CVF Conference on Computer Vision and Pattern Recognition, CVPR 2023, Vancouver, BC, Canada, June 17-24, 2023*, 2744–2753, DOI: 10.1109/CVPR52729.2023.00269 (IEEE, 2023).

5. Kim, E., Kim, S., Seo, M. & Yoon, S. Xprotonet: diagnosis in chest radiography with global and local explanations. In *Proceedings of the IEEE/CVF conference on computer vision and pattern recognition*, 15719–15728 (2021).
